# Bicyclic-ring base doping induces n-type conduction in carbon nanotubes with outstanding thermal stability in air

**DOI:** 10.1038/s41467-022-31179-6

**Published:** 2022-06-20

**Authors:** Shohei Horike, Qingshuo Wei, Kouki Akaike, Kazuhiro Kirihara, Masakazu Mukaida, Yasuko Koshiba, Kenji Ishida

**Affiliations:** 1 Department of Chemical Science and Engineering, Graduate School of Engineering, Kobe University, Kobe, Japan; 2 Nanomaterials Research Institute, National Institute of Advanced Industrial Science and Technology (AIST), Tsukuba, Japan; 3 PRESTO, Japan Science and Technology Agency, Kawaguchi, Japan; 4 Research Center for Membrane and Film Technology, Kobe University, Kobe, Japan

**Keywords:** Electron transfer, Thermoelectric devices and materials, Carbon nanotubes and fullerenes

## Abstract

The preparation of air and thermally stable n-type carbon nanotubes is desirable for their further implementation in electronic and energy devices that rely on both p- and n-type material. Here, a series of guanidine and amidine bases with bicyclic-ring structures are used as n-doping reagents. Aided by their rigid alkyl functionality and stable conjugate acid structure, these organic superbases can easily reduce carbon nanotubes. n-Type nanotubes doped with guanidine bases show excellent thermal stability in air, lasting for more than 6 months at 100 °C. As an example of energy device, a thermoelectric p/n junction module is constructed with a power output of *ca*. 4.7 μW from a temperature difference of 40 °C.

## Abstract (web summary)

The preparation of stable n-type carbon nanotubes is desirable for their potential applications in molecular electronics. Here, the authors report that doping with bicyclic-ring organic superbases enables the synthesis of n-type carbon nanotubes with excellent thermal stability in air.

## Introduction

Carbon nanotubes (CNTs) have attracted much attention as future components of electronic and energy devices, such as field-effect transistors^1^, photovoltaics^2^, terahertz scanners^3^, gas sensors^4^, and thermoelectric modules^5^. Their high charge carrier transport properties^6^, sharp features in the density of states (DOS)^7^, large specific surface area^8^, light weight^9^, and mechanical robustness and flexibility^10^ suggest the potential for realizing organic–inorganic hybrid or even fully molecular-based devices.

Importantly, these devices require both p- and n-type materials to create p/n junctions. For inorganic semiconductors, doping technology is well established today for modulating the material polarity^11^. Because the major charge carrier type and their density determine the material polarity and the corresponding device functions, the importance of doping is also recognized by researchers of molecular electronics^12^. Since the early days of CNT research, doping reagents and spectroscopic characterizations (*e.g*., Raman scattering^13–15^, absorption^16^, electron spin resonance (ESR)^17,18^, and photoelectrons^19^) have been extensively used to reveal the effects of doping.

Charge carrier doping in CNTs relies on charge transfer (redox reactions) between the CNT *π*-electron system and dopants on the surface or enclosed inside the nanotubes. Several dispersive interactions (*e.g*., *π*–*π*, *n*–*π*, *σ*–*π*, and ion–*π* interactions)^20,21^ can be responsible for the charge transfer. Electron accepting (oxidizing) and donating (reducing) materials induce p- and n-doped states in CNTs, respectively. p-Type doping of CNTs is rather easy because the CNTs readily accept holes from naturally adsorbed oxygen molecules in air^22^. In contrast, CNTs after n-type doping tend to lack air stability due to dopant volatilization^23^ and/or autoxidation of the electron-injected CNTs by oxygen in air. For example, alkali metal deposition on the CNT surface produces metal cation and available electron to promote a heavily n-doped state^24^; however, this n-type polarity could not be retained upon exposure to air. Much milder reducing chemicals such as nitrogen-containing molecules (*e.g*., tertiary amines and viologens)^25–28^, phosphines^29^, metallocene^30,31^, alkylammonium or imidazolium salts^32,33^, anionic surfactants^34^, and supramolecular salts^35^ could produce air-stable n-type CNTs that last several days to months. In addition to air stability (preservation stability), the thermal stability of n-type CNTs should also be improved for device applications. For instances, electric currents in logic circuits generate concentrated heat^36^, photovoltaic cells become hot under sunlight^37^, and thermoelectric modules need to function for long periods under elevated temperatures. A pioneering work was conducted by Nonoguchi and Kawai *et al*. using crown ether complexes as n-doping reagents for CNTs^35^.

Herein, we employ commercially available versatile molecular bases with bicyclic ring structures as electron donors for CNTs. This is inspired by the reported charge transfer between C_60_ and a bicyclic amidine base (1,8-diazabicyclo[5.4.0]-7-undecene, DBU)^38^, which is known as an organic superbase. Upon their mixing, fullerene could accept a single electron from DBU. Although that pioneering study did not examine the conducting properties of the product, it inspired us to apply this class of interaction between DBU and carbon *π*-electron systems to the electron doping of CNTs. Herein we also test bicyclic guanidine bases to further improve the n-type stability under elevated temperatures. Thermoelectric charge carrier analysis shows that doping with these bases successfully convert the as-prepared p-type CNTs into quite stable n-type via a facile solution-based method. Considering the excellent stability (over 6 months at 100 °C) of the n-type polarity, the simple doping process, and versatility in the base molecules, our results offer a potential avenue to produce n-type CNTs for future molecular electronics.

## Results

### Thermoelectric and work function characterization of doped CNTs

**Fig. 1:**
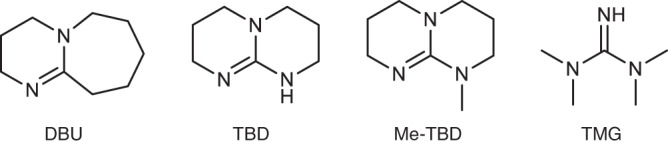
Molecular structures of dopants containing the amidine and guanidine moieties. DBU: 1,8-diazabicyclo[5.4.0]-7-undecene, TBD: 1,5,7-trizazabicyclo[4.4.0]dec-5-ene, Me-TBD: 7-methyl-1,5,7-triazabicyclo[4.4.0]dec-5-ene, TMG: 1,1,3,3-tetramethylguanidine.

Figure 1 depicts the chemical structures of the employed amidine and guanidine bases. 1,8-diazabicyclo[5.4.0]-7-undecene (DBU); 1,5,7-triazabicyclo[4.4.0]dec-5-ene (TBD); 7-methyl-1,5,7-triazabicyclo[4.4.0]dec-5-ene (Me-TBD); 1,1,3,3-tetramethylguanidine (TMG). These bases have strong basicity, showing much higher p*K*_a_ values (>20) in organic solvents than typical amines (at most 8)^39,40^. Thus, they are often considered organic superbases. The strong basicity has been explained from the viewpoints of (i) resonance stabilization of the conjugate acid, (ii) sp^2^-hybridization of the nitrogen atom, and (iii) a bicyclic ring that ties back the adjacent alkyl functionality, which reinforces the overlap between the lone-pair electrons of the sp^3^-hybridized nitrogen and the neighboring π* orbital, which leads to higher electron density and basicity of the sp^2^-hybridized (double-bonded) nitrogen for DBU, TBD, and Me-TBD^39^.

**Fig. 2:**
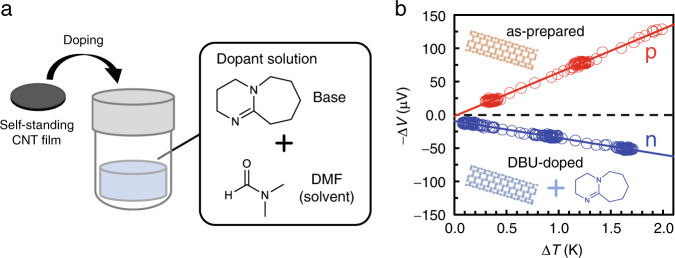
Fabrication of n-doped CNTs, and the thermopower of doped CNT films. **a** Schematic of doping by immersing the CNT films in a base solution. **b** Representative plots of thermopower (−Δ*V*) *vs*. the supplied temperature difference (Δ*T*) measured using the as-prepared (p-type) and DBU-doped (n-type) films made from EC1.5-CNT. Seebeck coefficients (*S*) were determined from the slopes in the plots (*S* = − Δ*V*/Δ*T*). Note that the potential of the lower temperature side was defined as ground.

Based on such strong basicity, we expect the efficient reduction of CNTs by using organic superbases. Single-walled CNTs (hereinafter denoted as EC1.5-CNT) were produced using enhanced direct injection pyrolytic synthesis (eDIPS)^41^. Self-standing CNT films (10–12 μm in thickness) with good electrical conductivity (680 S cm^−1^) and Seebeck coefficient (+60 μV K^−1^) were prepared by vacuum filtration. The doping process is schematically illustrated in Fig. 2a. The self-standing CNT films were immersed in the dopant solutions, and then dried in vacuo. Figure 2b shows representative measurement results used for determining the Seebeck coefficient (*S*) as the slope in thermopower (−Δ*V*) *vs*. the supplied temperature difference (Δ*T*). The sign of *S* reflects the major charge carrier type^42^, and the positive *S* for the as-prepared CNT film indicates p-type polarity (with holes as the major carrier species), possibly due to autoxidation in ambient air^22^. Figure 2b shows the n-type conversion of the CNT films upon doping with DBU; *S* changes from positive to negative (−24 μV K^−1^) after immersion in a *N,N*-dimethylformamide (DMF) solution of DBU with a concentration of 545 mM at ~298 K for 5 min and drying under vacuum.

**Fig. 3:**
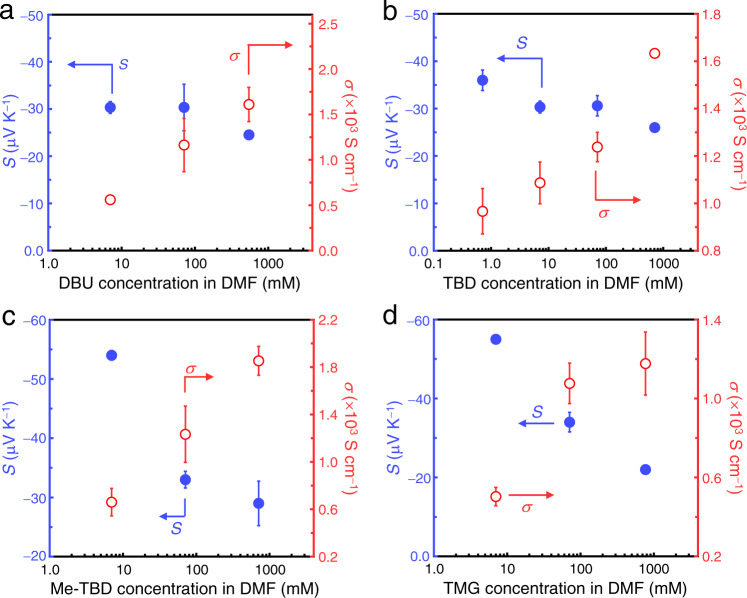
Dopant-concentration-dependent Seebeck coefficient (*S*) and electrical conductivity (*σ*). n-Type specimens were prepared by immersing EC1.5-CNT films in DMF solutions of **a** DBU, **b** TBD, **c** Me-TBD, and **d** TMG with varied concentrations at approximately 298 K for 5 min. Closed and open circles indicate *S* and *σ*, respectively. Data statistics: *n* ≥ 3. Error bars indicate the corresponding standard deviations (SD).

As shown in Fig. 3a–d, doping with any of the aforementioned bases produced n-type CNTs with *S* ≈ − 20 to −55 μV K^−1^ and good-to-excellent electrical conductivity (500–1800 S cm^−1^). The Seebeck coefficient and electrical conductivity tend to decrease and increase, respectively, with the concentration of the dopant solutions, reflecting the complementary relation between these parameters^43^. In comparison, immersing the CNT film in pure DMF hardly changed the value of *S* (+59 μV K^−1^), much less its sign. Thus, the proposed bases can reduce CNTs and successfully alter the major charge carrier from holes to electrons. Further, it should be noted that the doping was completed immediately after immersing the film in the dopant solution. Long-time (19 h) immersion of the EC1.5-CNT film in the DMF solution of TBD (71 mM) did not affect the resultant n-type properties; *S* = − 28 μV K^−1^ and *σ* = 1210 S cm^−1^ were obtained, both of which are similar to those of the films that were immersed only for 5 min. We also varied the temperature during the doping process of TBD in DMF (71 mM) using EC-1.5 CNT films: both hot (423 K) and cold (261 K) conditions resulted in negative Seebeck coefficients (−32 and −35 μV K^−1^, respectively) and high electrical conductivities (1620 and 1350 S cm^−1^, respectively). These values are similar to those of TBD-doped CNT films at room temperature (~298 K) as shown in Fig. 3b (we prevented photoirradiation during immersion in the dopant solution by wrapping an aluminum foil around the vial; therefore, supplying excess energy is not required for the electron transfer reactions). The processability of such low temperature (that is, low-energy cost) and fast reaction rate to prepare n-type CNTs should be considered. Recently, 1*H*-benzoimidazole derivatives have been used as n-type dopants, but they should be subjected to annealing in order to react with nanoscale carbon materials and organic semiconductors^44,45^. In contrast, the dopants reported here can be used without additional heating and thus, can be conceptualized as ready-to-use electron donors for nanoscale carbon materials.

**Fig. 4:**
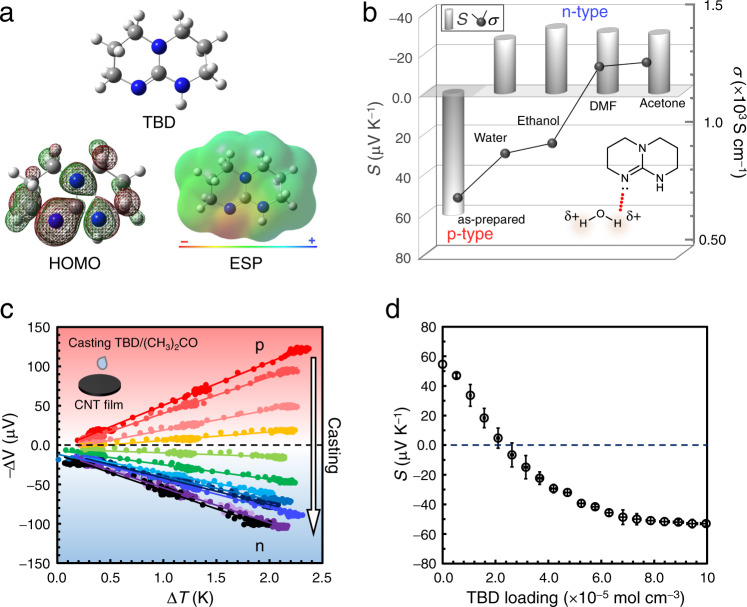
Calculated electronic structure of TBD, effects of different solvents, and stepwise doping of TBD to CNT. **a** Molecular surface mapping of TBD from DFT (B3LYP/6-31 G) calculation. White, gray, and blue balls indicate hydrogen, carbon, and nitrogen atoms, respectively. Red and green colors indicate positive and negative phases of the orbital, respectively. ESP: electrostatic potential. Warm color indicates a more negative potential compared to the other locations. Calculation results for the other bases are shown in Supplementary Fig. 1 of SI. For all dopants, the double-bonded nitrogen atom participates in the HOMO and carries high negative charge, suggesting dominant contribution of this site towards electron transfer to CNTs. **b** Variation in Seebeck coefficient (*S*) and electrical conductivity (*σ*) of TBD-doped CNT films according to the type of solvents. TBD concentration in DMF was fixed at 71 mM (10 mg mL^−1^). Inset: schematic interaction between lone-pair electrons of TBD and acidic hydrogen of water (a polar protic solvent). Raw data of solvent dependencies are summarized in Supplementary Table 1. **c** Representative plots of thermopower (−Δ*V*) *vs*. the supplied temperature difference (Δ*T*) for CNT films doped with given amounts of TBD. Inset: schematic of the doping process, in which the TBD loading was controlled by the drop-casting of given volumes of TBD solution in acetone (0.17 g L^−1^) on the CNT film (*ϕ* 17 mm, 11.6 μm in thickness). After repeated doping, the thermopower slope finally displays n-type polarity. **d** Seebeck coefficient of the CNT films (slope of the plots in panel c) according to the molar loading of TBD. It is estimated that *S* = 0 μV K^−1^ at a TBD loading of ~2.2 × 10^−5^ mol cm^−3^. Data statistics: *n* ≥ 3. Error bars indicate the corresponding SDs.

Similar to many amine compounds such as polyethyleneimine, the lone-pair electrons on the nitrogen atoms of doped amidine and guanidine would contribute to the electron injection into the CNTs (*n*–π* interaction)^46^. Density functional theory (DFT) calculation reveals that the double-bonded nitrogen atom of TBD participates in the highest occupied molecular orbital (HOMO) and carries a high negative charge (Fig. 4a), suggesting that the lone-pair electrons on this nitrogen atom mainly contribute to the CNT reduction^47^. Similar calculation results were also obtained for DBU, Me-TBD, and TMG (Supplementary Fig. 1 of Supplementary Information (SI)). This result is consistent with the mechanisms for the strong basicity of these compounds as proposed above^39^.

The lone pair donation from TBD to CNT was also supported by the solvent-dependent thermoelectric properties of doped CNTs. Figure 4b displays the variations in Seebeck coefficient and electrical conductivity of CNT films after TBD doping in polar protic (water and ethanol) and polar aprotic (DMF and acetone) solvents, and the respective molecular structures are shown in Supplementary Fig. 2 of SI. For all solvents, the sign of Seebeck coefficient becomes negative, indicating n-type polarity. Meanwhile, the conductivities are remarkably high (~1200 S cm^−1^) when using polar aprotic solvents, while those obtained using polar protic solvents are at most 900 S cm^−1^. The electrical conductivity is generally proportional to the charge carrier density^43^, and it is evident that TBD has a weaker reducing ability in polar protic solvents than in aprotic ones. By analogy with nucleophilic reactions in organic chemistry (*e.g*., S_N_2 reaction)^48^, solvation of the nucleophiles should affect electron transfer from the bases to CNTs. The acidic hydrogen atoms of polar protic solvents could accept lone pairs of electrons to form hydrogen bonds (schematically illustrated in the inset of Fig. 4b), while the aprotic solvents lack such highly polarized hydrogen. Therefore, the lower electron donating ability of TBD in protic solvents would be attributed to suppression of electron transfer to CNTs by the surrounding solvent molecules. This comparison further supports the lone-pair electron donations as the mechanism of n-type doping of CNTs by the presented molecular bases.

It is noteworthy that electron injection into the CNTs using TBD can be controlled stepwise according to the Seebeck coefficients measured for different amounts of TBD loading (controlled by drop-casting a certain volume of TBD/acetone solution of known concentration). Figure 4c and d show respectively the thermopower evaluation results and the Seebeck coefficients according to TBD loading. The initially positive Seebeck coefficient gradually declines, reaches ~0 μV K^−1^ at a TBD density of ~2.2 × 10^−5^ mol cm^−3^ (calculated from the molar amount of loaded TBD per volume of CNT film), and then becomes negative at a higher loading. The low-energy continuous charge carrier modulation by the wet-process will be important for tuning the device function in future molecular electronics.

**Fig. 5:**
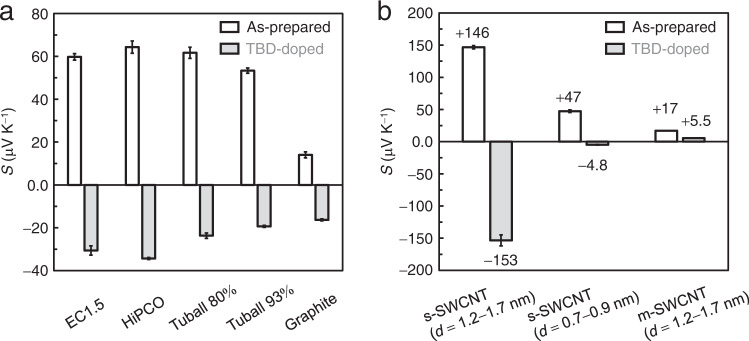
Feasibility of TBD doping on different nanoscale carbon materials in term of Seebeck coefficient (*S*) measured at room temperature (~298 K) in air. **a** Variations in Seebeck coefficient of different nanoscale carbon materials. Doping was performed using DMF solution of TBD (71 mM). The doping condition was the same as that for single-walled CNTs synthesized using the eDIPS method (EC1.5, Meijo Nano Carbon), which is described in detail in METHOD section. The percentages (80% and 93%) of the Tuball CNTs indicate purity of the pristine materials. Data statistics: *n* ≥ 3. Error bards indicate (SD). **b** Variations in Seebeck coefficient according to the conducting type of SWCNTs. Data statistics: *n* = 3. Error bars indicate the corresponding SDs.

TBD was used to investigate the feasibility of the dopants, as well as the facile solution-based doping process for a wider range of nanoscale carbon materials. In Fig. 5a, films of CNTs (without sorting of metallic and semiconducting nanotubes) furnished by other synthesis methods (HiPCO and Tuball) and graphite were all successfully converted to n-type by immersing into the DMF solution of TBD (71 mM) at ∼298 K for 5 min (changes of electrical conductivity can be seen in Supplementary Fig. 3 of SI). However, we found that the n-type conversion of CNTs by TBD doping has significant dependences on the conducting type and tube diameter. We subsequently investigated the feasibility of superbase doping by performing the experiment using semiconducting SWCNTs (s-SWCNTs) (i) with a tube diameter of 1.2–1.7 nm, (ii) a mean tube diameter of 0.7–0.9 nm (s-SWCNTs with (6,5) chirality index-enriched sample; tube diameter of ∼0.75 nm), and (iii) metallic SWCNTs (m-SWCNTs) with a tube diameter of 1.2–1.7 nm. Doping was performed by immersing the films in TBD solution (71 mM) at ~298 K for 5 min. Similar to the non-separated CNT samples, the as-prepared films of these sorted CNTs exhibited positive Seebeck coefficients ((i) +146, (ii) +47, and (iii) +17 μV K^−1^), as shown in Fig. 5b. Changes in the coefficients upon doping differed significantly according to the conducting types of nanotubes, which is also shown in Fig. 5b. (i) s-SWCNTs (1.2–1.7 nm in diameter) were converted into n-type with a negative Seebeck coefficient of −153 μV K^−1^. (ii) s-SWCNTs (0.7–0.9 nm in diameter) were also converted into n-type, but the coefficient was close to zero (only − 4.8 μV K^−1^). For (iii) m-SWCNTs, the coefficient decreased to close to zero (+5.5 μV K^−1^) but did not change to a negative value. Considering these changes in the coefficients, it is apparent that TBD can reduce each class of CNT. Meanwhile, this effect significantly depends on the conducting type and tube diameter.

The variation in reducing ability of TBD depending on the nanotube diameters and conducting types could be also recognized from the difference in the shifts in the nanotube work functions after doping. Table 1 lists the shift measured using Kelvin probe method (described in more detail in Supplementary Fig. 4 of SI) and first interband transition energy of each CNT sample calculated using the following equations (the energy is represented by *E*(S_11_) and *E*(M_11_), where S_11_ and M_11_ represent interband transitions in s- and m-SWCNT, respectively)^49^:

1 $$E\left({{{{{{\rm{S}}}}}}}_{11}\right)=\frac{0.962}{d},$$

2 $$E\left({{{{{{\rm{M}}}}}}}_{11}\right)=\frac{2.60}{d},$$

where *d* is the diameter of SWCNT. Note that *E*(S_11_) can be regarded as the bandgap energy. After doping with TBD, the work functions of all CNT samples decreased by several hundreds of meV (Table 1), confirming the reduction of CNTs with TBD doping.

**Table 1:** Work function shifts upon doping of TBD according to the conducting type of SWCNTs measured in air.

Sample	Work function shift (eV)	*E*(S_11_) or *E*(M_11_)^†^ (eV)
s-SWCNT (*d* = 1.2−1.7 nm)	−0.74	0.56−0.80
s-SWCNT (*d* = 0.7−0.9 nm)	−0.61	1.06−1.37
m-SWCNT (*d* = 1.2−1.7 nm)	−0.61	1.52−2.16
EC1.5	−0.30	—

^†^*E*(S_11_) and *E*(M_11_): first interband transition energy of semiconducting and metallic SWCNTs.

TBD doping was performed by immersing the CNT films in the acetone solution of TBD with a concentration of 71 mM. First interband transition energy is calculated using equations reported elsewhere^49^.

The work function shift for the s-SWCNTs (0.7–0.9 nm in diameter) and m-SWCNTs (1.2–1.7 nm in diameter) after doping (0.61 eV) was approximately half or one third of *E*(S_11_) or *E*(M_11_) of these CNTs, respectively. Therefore, these results confirmed that the electron injection into either LUMO of the s-SWCNTs (0.7–0.9 nm in diameter) or the π^*^ band of the m-SWCNTs (1.2–1.7 nm in diameter) was hardly achieved by TBD doping. These observations are consistent with the changes of the Seebeck coefficients close to zero. For the s-SWCNTs (1.2–1.7 nm in diameter), the decrease of 0.74 eV is nearly identical to the interband transition energy (*i.e*., bandgap energy of 0.56−0.80 eV); thus, the electron injection into the LUMO of the s-SWCNTs is confirmed. This confirmation directly corresponds to the clear positive-to-negative conversion of the Seebeck coefficient for this sample shown in Fig. 5b. The LUMO levels of the s-SWCNTs with a larger diameter of 1.2–1.7 nm could be deeper than that of the SWCNT with (6,5) chirality index^50^, and therefore, an electron can be easily injected from TBD to the LUMO.

With respect to the non-separated CNT samples (EC1.5, HiPCO, and Tuball) that are the mixtures of s- and m-SWCNTs with different diameters and chiralities, two scenarios can be considered for explaining the induction of overall negative Seebeck coefficients: (1) semiconducting CNTs (and metallic CNTs) with tube diameters feasible for accepting electron injection from TBD become n-type with relatively large negative Seebeck coefficients, while the other CNTs with less accessibility to doping exhibit small coefficients close to zero, thereby resulting in the overall material exhibiting a negative Seebeck coefficient. (2) Previous papers on chemical doping of conducting polymers proposed that carrier injection occurs not only from the dopant to the polymer but also within and between the polymer molecules^51,52^. An electron may be initially injected into the CNTs that can easily accept electrons into their LUMO levels and then get transferred via CNT/CNT intermolecular interactions to yield n-doped state to the whole film. Nevertheless, the possibility of this should be further examined using CNTs with a variety of separated metallic/semiconducting tubes with various diameters and chiralities.

### Thermal stability of created N-type materials

**Fig. 6:**
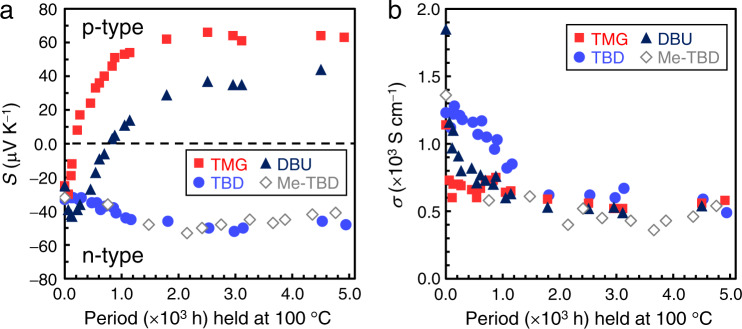
Long-term stability of n-doped EC1.5-CNT films during incubation at 100 °C in air. **a** Changes in Seebeck coefficient (*S*). **b** Changes in electrical conductivity (*σ*). Both parameters were measured along the in-plane direction of the film. All measurements were performed at 25 °C in air. Specimens were prepared by immersing EC1.5-CNT buckypapers in the DMF solutions of DBU, TBD, Me-TBD, and TMG with concentrations of 545, 71, 71, and 769 mM, respectively.

To examine the thermal stability of the n-type polarity for doped CNT films, their Seebeck coefficient and electrical conductivity were measured after heating at 100 °C in air. As shown in Fig. 6, thermal stability of the n-doped CNT materials in air changes significantly according to the primary molecular structures of applied bases. The n-type CNT films created by TMG and DBU doping seem to have low thermal stability. The former readily reverted to p-type after 222 h at 100 °C (Fig. 6a), and its electrical conductivity decreased to approximately 500 S cm^−1^ (Fig. 6b). A positive Seebeck coefficient (*i.e*., with holes as the major charge carrier species) means that the n-type CNTs suffered dedoping (autoxidation) upon heating in air^22^. For the DBU-doped CNT film, the specimen remained relatively stable compared to the TMG-doped CNT, but its n-type polarity was not well retained. The Seebeck coefficient changed to −6 μV K^−1^ after heating for 696 h (~1 month) and then completely reverted to p-type after further heating (Fig. 6a). Similarly, the electrical conductivity monotonically decreased, declining by 70% during this heating period (Fig. 6b).

On the other hand, TBD- and Me-TBD-doped CNT films exhibited higher stability than TMG- and DBU-doped nanotubes under the same heating condition, as their Seebeck coefficients remained negative after ∼5,000 h (over half a year) as shown in Fig. 6a. We believe this is one of the highest thermal stabilities in air reported for n-type CNT materials. After ~2,000 h of heating, slight increases of the negative Seebeck coefficient (up to −50 μV K^−1^) and the decreases in electrical conductivity (shown in Fig. 6b) may reflect partial dedoping of the n-doped CNTs. Nevertheless, the Seebeck coefficients plateaued at approximately −50 μV K^−1^ during further heating. As electronic and energy devices invariably become heated during operation^36,37^, it is crucial to preserve not only the physical stability of the materials but also the thermal stability of n-type doping. Although some research groups have created n-type CNT materials stable in air near room temperature, the stability at elevated temperature has not been tested adequately. Several reported Seebeck measurements of n-doped CNTs at elevated temperatures were conducted under inert He gas atmosphere instead of air^23,53^. To the best of our knowledge, only the study by Nonoguchi and Kawai *et al*. examined the thermal stability in air of n-type CNTs produced with supramolecular salts^35^. Therefore, the versatile bicyclic guanidine bases as well as the simple doping process presented in this study are innovative for manufacturing n-type CNTs suitable for device applications. The stability differences according to the primary molecular structures of the applied bases may be attributed to the differences of (i) adsorption ability onto the nanotube surface (volatilization), (ii) the charge balances between the negative charges introduced in the CNTs and the positive charge in the conjugate acid state of the base to stabilize the n-doped state of CNTs^35^, and (iii) the blocking ability of oxygen impurity from ambient air that may cause dedoping, and these possibilities should be investigated in future works. Revealing such substantial mechanisms for the n-type stabilization will also contribute to the development of fully molecular-based devices. Our comparisons of the base molecular structures indicate that bicyclic-ring bases with guanidine moiety are useful reagents for creating n-type CNTs that are thermally stable in air.

### Demonstration of CNT-based thermoelectric energy device

We employed thermoelectric measurement to elucidate the major carrier type in CNTs before and after doping. Thermoelectric materials could also be used in waste heat recovery and/or energy harvesting systems for powering wireless sensors in future Internet of Things^54^. Pairing the as-prepared p-type CNTs with n-doped counterparts is one promising way to manufacture molecular-based thermoelectric generators with high power outputs^55^. This potential application of thermally stable n-type CNTs is demonstrated here in an all-CNT thermoelectric module.

**Fig. 7:**
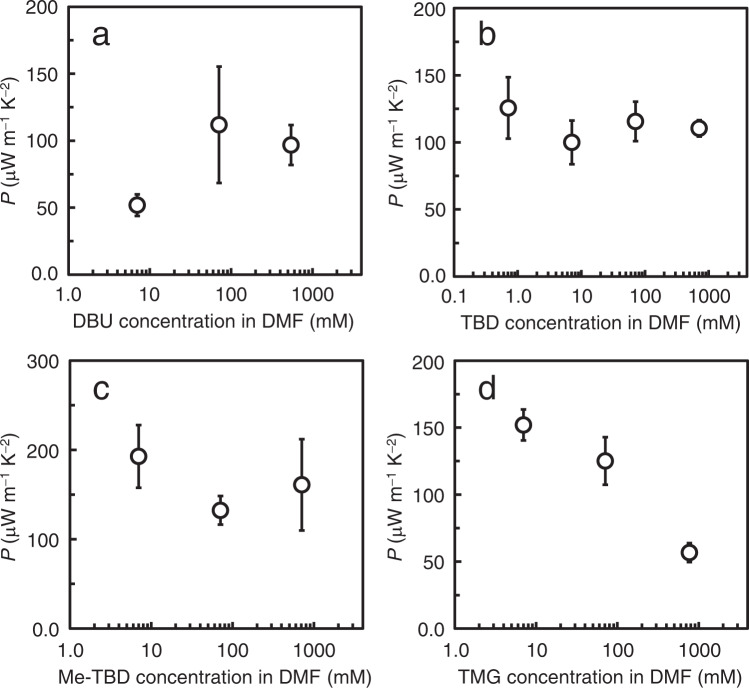
Dopant-concentration-dependent Power factor (*P*). n-Type specimens were prepared by immersing EC1.5-CNT films in DMF solutions of **a** DBU, **b** TBD, **c** Me-TBD, **d** TMG with varied concentrations. Data statistics: *n* ≥ 3. Error bars indicate the corresponding SDs. The ideal variations of thermoelectric power factor according to carrier density (taking maximum value at the specific carrier density), similar to that in typical semiconducting thermoelectric materials, is not observed in the present material systems, which would be because the used CNT sample (EC1.5-CNT) is a mixture of semiconducting and metallic nanotubes with various diameters and chirality.

The performance of thermoelectric materials is evaluated by the power factor (*P* = *S*^2^*σ*) and dimensionless figure of merit (*ZT* = *S*^2^*σT*/*κ*), where *κ* is the thermal conductivity^56^. The power factor of each specimen is readily calculated from the Seebeck coefficient and electrical conductivity (Fig. 7). For the considered bases (DBU, TBD, TMG, and Me-TBD), TBD- and Me-TBD-doped CNT films provide the highest power factors (over 100 μW m^−1^ K^−2^), regardless of the dopant concentration in the DMF solutions. For TBD doping, higher power factors were obtained using polar aprotic solvents than protic ones (see Supplementary Table 1 of SI) because of the higher electrical conductivities as discussed in Fig. 4b. Therefore, we chose CNT films doped with DMF solution of TBD for further analysis and module preparation. The thermal conductivity was determined from the thermal diffusivity, heat capacity, and density of the films as shown in Table 2. Upon doping, the thermal diffusivity slightly decreases from 52 to 38 mm^2^ s^−1^, possibly due to phonon scattering by TBD molecules on the CNT surface and/or CNT/CNT junctions. However, due to the almost constant heat capacity (from 0.71 to 0.78 J g^−1^ K^−1^) and the slightly increased density (from 1.1 to 1.3 g cm^−3^), the thermal conductivity of the doped film was nearly identical to that of the as-prepared CNT film (38 and 40 W m^−1^ K^−1^, respectively). Consequently, the *ZT* value (Table 2) of the TBD-doped film is lower than that of the as-prepared film due to the lower power factor. Recent studies revealed that carefully sorted semiconducting CNTs may reach higher *P* and/or *ZT* than metallic ones and their mixtures^57^, which is a possible strategy to further improve the thermoelectric performance.

**Table 2:** Thermal properties and dimensionless figure of merits for as-prepared and TBD-doped EC1.5-CNT films measured at 298 K in air. TBD doping was performed by immersing the CNT film in the DMF solution of TBD with a concentration of 71 mM.

Sample	*a*^†^ (mm^2^ s^−1^)	*C*_p_^‡^ (J g^−1^ K^−1^)	*ρ*^§^ (g cm^−3^)	*κ*^⊥^ (W m^−1^ K^−1^)	*ZT*^//^ (at 298 K)
As-prepared	52	0.71	1.1	40	1.8 × 10^−3^
TBD-doped	38	0.78	1.3	38	9.0 × 10^−4^

^†^*a*: in-plane thermal diffusivity; ^‡^*C*_p_: heat capacity; ^§^*ρ*: density; ^⊥^*κ*: in-plane thermal conductivity; ^//^*ZT*: dimensionless figure of merit in the in-plane direction.

**Fig. 8:**
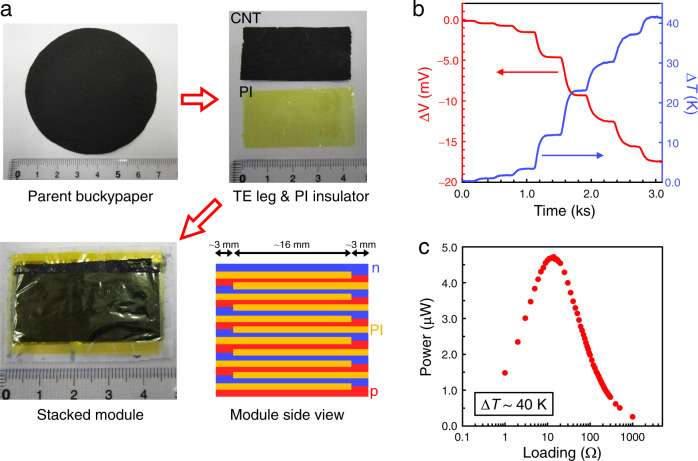
Demonstration of a CNT-based TE module. **a** Photographs and schematic. A large piece of parent buckypaper (*ϕ* ≈ 73 mm) was produced and cut into a strip of 22 mm × 43 mm. The as-prepared CNTs were used for the p-type TE legs. For the n-type ones, the CNTs were doped using DMF solution of TBD (71 mM). Then, the p- and n-type CNT legs were alternatively stacked and electrically connected at the edges (~3 mm), while polyimide (PI) films (23 mm × 44 mm) were inserted between them for insulation. The module is composed of 6 pairs of p/n couples. **b** Temporary change in the open circuit voltage (Δ*V*) for the module according to the supplied temperature difference (Δ*T*). The thermopower of the 6-couple module is *ca*. 415 μV K^−1^. **c** Power output from the module at Δ*T* of ~40 K under varied load resistance.

We constructed an all-CNT thermoelectric module consisting of 6 p/n couples, by alternatively stacking p- and n-type CNT films and inserting insulating polyimide (PI) films between them (Fig. 8a). This connection (electrically in series and thermally in parallel) enables efficient addition of thermopower from each component. The thermopower generated by the module is *ca*. 415 μV K^−1^ according to the open circuit voltage *vs*. temperature difference (Δ*T*) data in Fig. 8b. At Δ*T* = 40 K, the open circuit voltage reached ~17 mV, and the maximum power output of 4.7 μW was obtained after loading a resistance of 14 Ω, as shown in Fig. 8c. Based on the cross-sectional area of the module (0.0153 cm × 4.3 cm), the maximum power density is 71 μW cm^−2^, which is good-to-excellent compared to previous all-organic thermoelectric modules. Some of the reported values are: 167 μW cm^−2^ at Δ*T* = 27.5 K (all-CNT thermoelectric module)^58^, 0.27 μW cm^−2^ at Δ*T* = 30 K (all-organic module)^59^, 15 nW cm^−2^ at Δ*T* = 45 K (all-polymer disk-shaped module)^60^, and 40 μW cm^−2^ at Δ*T* = 50 K (natural cooling condition) using a polymer/nickel module in our recent study^61^. Thermoelectric devices for energy harvesting applications face competition from Li-ion batteries with high energy densities and long lifetimes. The TBD- (or Me-TBD) doping technique developed here would contribute to the long-term operation of all-CNT thermoelectric modules. Further, the power factor of CNT legs may be improved by using recent synthetic techniques^62,63^ or sorted semiconducting CNTs^57^. In these contexts, this study contributes to developing all-CNT thermoelectric devices for thermal energy recovery.

In summary, organic superbases were successfully applied to convert as-prepared p-type CNTs into n-type ones with good thermal stability in air, negative Seebeck coefficients, and good-to-excellent electrical conductivities. Efficient electron transfer from bicyclic-ring guanidine to CNTs was achieved even near room temperature, and the resultant n-type polarity persisted for more than 6 months at 100 °C in air. The present results highlight the potential of molecular structure design for exploring and developing doping reagents because the stability of n-type CNTs is significantly affected by minor changes in the primary structure of dopant molecule. Future studies are needed to investigate the origin of these differences in stability. The demonstrated excellent thermal stability could facilitate development of molecular devices such as thermoelectric energy generators, as exemplified in this study.

## Methods

### Film formation and doping method

The chemicals and CNT samples used and their suppliers are summarized in Supplementary Tables 2 and 3 of SI. All chemicals were used as purchased. The CNTs were roughly dispersed at approximately 0.1 mg mL^−1^ in an aqueous solution of Brij 30 nonionic surfactant (0.8 mg mL^−1^) in a 50 mL screw vial using an ultrasonic homogenizer (Q500, QSONICA) in an ice bath. The CNT dispersion was vacuum-filtered on a polytetrafluoroethylene membrane with a pore size of 0.2 μm (H020A025A, ADVANTEC). The resultant buckypaper was peeled off, rinsed with water and acetone, and then dried in air overnight. The obtained circular self-standing CNT film samples were 17 mm in diameter and 10–12 μm in thickness.

Dopant solutions were prepared using a vortex mixer in 30 mL screw vials. Doping was typically conducted by immersing the CNT films in the solutions, and then the films were dried under vacuum for 30 min. To control the amount of doping (experiments shown in Fig. 4c, d), an acetone solution of TBD (0.17 g L^−1^) was prepared, and 10–12 μL of the solution was drop-casted onto the CNT film. After drying in vacuo for 15 min, the Seebeck measurement was conducted.

### Characterizations

The test environment was air throughout the measurements. Thicknesses of the CNT films were measured by differential transformer principle using ai-Phase M3 (ai-Phase) apparatus. For the Seebeck measurements, the specimen was placed on two Peltier modules (VPE20-30S, VICS) to apply a temperature difference. R-type thermocouples were directly attached to the CNT film to simultaneously measure the voltage and the temperature using a data logger (LR8400, HIOKI). The potential at the lower temperature side was defined as ground. Electrical conductivities of CNT films were measured by four-probe method using a resistivity meter (Loresta-GP MCP-T610, MITSUBISHI CHEMICAL ANALYTECH). The Seebeck coefficient and electrical conductivity were determined as the averages over more than 3 repeated measurements.

To investigate the stability of n-type polarity, the Seebeck coefficients and electrical conductivities of doped CNT films were measured after storing for a given period at 100 ± 0.3 °C in air within an incubator (SU-222, ESPEC). No passivation was applied to the films throughout the heating and measurements. Note that the measurements were conducted in room-temperature air (approximately 298 K) after removing samples from the incubator. In-plane thermal diffusivity was measured according to the periodic heating radiation thermometry technique (see Supplementary Fig. 5 of SI) using a contactless thermal diffusivity measurement system (Thermowave Analyzer TA35, BETHEL). Heat capacity of the as-prepared and TBD-doped CNTs was measured using differential scanning calorimetry (DSC 200 F3 Maia, NETZSCH) under N_2_ gas flow (50 mL min^−1^). The density of the CNT films was calculated from the sample weight and dimension. In-plane thermal conductivities (*κ*) of the CNT films were calculated from the equation *κ* = *aC*_p_*ρ*, where *a* is the thermal diffusivity, *C*_p_ is the heat capacity, and *ρ* is the density. Work function shift of the CNT samples was performed in ambient air using a commercial single-point Kelvin probe system (KP020, KP Technology) with the resolution of 1−3 meV.

### Quantum calculations

The structural optimization, molecular orbitals, and electrostatic potentials of the dopants were calculated by DFT (B3LYP/6-31 G) using Gaussian 09 package.

## Supplementary information

Supplementary Information

## Data Availability

The authors confirm that the data supporting the findings of this study are available within the article and its supplementary material (molecular mapping of dopants; chemical structures of solvents; electrical conductivity of TBD-doped CNT and graphite films; measurement principle of work function shift using Kelvin probe; raw data of thermoelectric measurements; list of chemicals and materials; thermal diffusivity). Source data that support the plots within this paper are provided with this paper. Source data are provided with this paper.

## Source data

Source data file

## References

[1] Tans SJ, Verschueren ARM, Dekker C (1998). Room-temperature transistor based on a single carbon nanotube. Nature.

[2] Wu ZC (2004). Transparent, conductive carbon nanotube films. Science.

[3] Suzuki D, Oda S, Kawano Y (2016). A flexible and wearable terahertz scanner. Nat. Photonics.

[4] Han T, Nag A, Mukhopadhyay SC, Xu YZ (2019). Carbon nanotubes and its gas-sensing applications: a review. Sens. Actuator A Phys..

[5] Yao Q, Chen LD, Zhang WQ, Liufu SC, Chen XH (2010). Enhanced thermoelectric performance of single-walled carbon nanotubes/polyaniline hybrid nanocomposites. ACS Nano.

[6] Durkop T, Getty SA, Cobas E, Fuhrer MS (2004). Extraordinary mobility in semiconducting carbon nanotubes. Nano Lett..

[7] Mintmire JW, White CT (1998). Universal density of states for carbon nanotubes. Phys. Rev. Lett..

[8] Chakraborty S (2006). Surface area measurement of functionalized single-walled carbon nanotubes. J. Phys. Chem. B.

[9] Laurent C, Flahaut E, Peigney A (2010). The weight and density of carbon nanotubes versus the number of walls and diameter. Carbon.

[10] Coleman JN, Khan U, Gun’ko YK (2006). Mechanical reinforcement of polymers using carbon nanotubes. Adv. Mater..

[11] Jones KS, Lind AG, Hatem C, Moffatt S, Ridgway MC (2013). A brief review of doping issues in III-V semiconductors. ECS Trans..

[12] Chiang CK (1978). Conducting polymers: halogen doped polyacetylene. J. Chem. Phys..

[13] Rao AM, Eklund PC, Bandow S, Thess A, Smalley RE (1997). Evidence for charge transfer in doped carbon nanotube bundles from Raman scattering. Nature.

[14] Das A (2007). Doping in carbon nanotubes probed by raman and transport measurements. Phys. Rev. Lett..

[15] Grimm S (2017). Doping-dependent G-mode shifts of small diameter semiconducting single-walled carbon nanotubes. Carbon.

[16] Kazaoui S, Minami N, Matsuda N, Kataura H, Achiba Y (2001). Electrochemical tuning of electronic states in single-wall carbon nanotubes studied by in situ absorption spectroscopy and ac resistance. Appl. Phys. Lett..

[17] Chauvet O (1996). ESR study of potassium-doped aligned carbon nanotubes. Phys. Rev. B.

[18] Matsumoto D, Yanagi K, Takenobu T, Okada S, Marumoto K (2015). Electrically induced ambipolar spin vanishments in carbon nanotubes. Sci. Rep..

[19] Suzuki S, Bower C, Watanabe Y, Zhou O (2000). Work functions and valence band states of pristine and Cs-intercalated single-walled carbon nanotube bundles. Appl. Phys. Lett..

[20] Mutai K, Kobayashi K, Kobayashi T, Utsunomiya C (1977). Intramolecular charge-transfer interaction between lone pair electrons and π-electron system. Chem. Lett..

[21] Schneider H-J (2015). Dispersive interactions in solution complexes. Acc. Chem. Res..

[22] Kang D, Park N, Ko JH, Bae E, Park W (2005). Oxygen-induced p-type doping of a long individual single-walled carbon nanotube. Nanotechnology.

[23] Hata S (2020). Development of carbon nanotube organic thermoelectric materials using cyclodextrin polymer: control of semiconductor characteristics by the solvent effect. Jpn. J. Appl. Phys..

[24] Lee RS, Kim HJ, Fischer JE, Thess A, Smalley RE (1997). Conductivity enhancement in single-walled carbon nanotube bundles doped with K and Br. Nature.

[25] Shim M, Javey A, Kam NWS, Dai HJ (2001). Polymer functionalization for air-stable n-type carbon nanotube field-effect transistors. J. Am. Chem. Soc..

[26] Sarabia-Riquelme R (2017). Simple, low-cost, water-processable n-type thermoelectric composite films from multiwall carbon nanotubes in polyvinylpyrrolidone. Synth. Met..

[27] Hata S (2019). Highly-stable n-type carbon nanotube material under accelerated aging conditions: conjunctive effect of hydrazine derivatives and commodity polymers. Chem. Lett..

[28] Kim SM (2009). Reduction-controlled viologen in bisolvent as an environmentally stable n-type dopant for carbon nanotubes. J. Am. Chem. Soc..

[29] Nonoguchi Y (2013). Systematic conversion of single walled carbon nanotubes into n-type thermoelectric materials by molecular dopants. Sci. Rep..

[30] Shiozawa H (2008). A catalytic reaction inside a single-walled carbon nanotube. Adv. Mater..

[31] Li XK (2014). Controlled doping of carbon nanotubes with metallocenes for application in hybrid carbon nanotube/Si solar cells. Nano Lett..

[32] Cheng XJ, Wang X, Chen GM (2018). A convenient and highly tunable way to n-type carbon nanotube thermoelectric composite film using common alkylammonium cationic surfactant. J. Mater. Chem. A.

[33] Horike S, Wei QS, Kirihara K, Mukaida M (2020). Water-processable n-type doping of carbon nanotubes via charge transfer with imidazolium chloride salt. Chem. Phys. Lett..

[34] Seki Y, Nagata K, Takashiri M (2020). Facile preparation of air-stable n-type thermoelectric single-wall carbon nanotube films with anionic surfactants. Sci. Rep..

[35] Nonoguchi Y (2016). Simple salt-coordinated n-type nanocarbon materials stable in air. Adv. Funct. Mater..

[36] Wei J (2008). Challenges in cooling design of CPU packages for high-performance servers. Heat. Transf. Eng..

[37] Royne A, Dey CJ, Mills DR (2005). Cooling of photovoltaic cells under concentrated illumination: a critical review. Sol. Energy Mater. Sol. Cells.

[38] Skiebe A, Hirsch A, Klos H, Gotschy B (1994). [DBU]C_60_. Spin pairing in a fullerene salt. Chem. Phys. Lett..

[39] Hyde AM, Calabria R, Arvary R, Wang X, Klapars A (2019). Investigating the underappreciated hydrolytic instability of 1,8-Diazabicyclo[5.4.0]undec-7-ene and related unsaturated nitrogenous bases. Org. Process Res. Dev..

[40] Geiselhart CM, Schmitt CW, Jockle P, Mutlu H, Barner-Kowollik C (2019). A guanidine-based superbase as efficient chemiluminescence booster. Sci. Rep..

[41] Saito T (2008). Selective diameter control of single-walled carbon nanotubes in the gas-phase synthesis. J. Nanosci. Nanotechnol..

[42] Sun PJ (2015). Large Seebeck effect by charge-mobility engineering. Nat. Commun..

[43] Tan JJ (2019). Balancing the electrical conductivity and Seebeck coefficient by controlled interfacial doping towards high performance benzothienobenzothiophene-based organic thermoelectric materials. J. Mater. Chem. A.

[44] Wei P, Oh JH, Dong GF, Bao ZN (2010). Use of a 1*H*-benzoimidazole derivative as an *n*-type dopant and to enable air-stable solution-processed *n*-channel organic thin-film transistors. J. Am. Chem. Soc..

[45] Liu Y, Villalva DR, Sharma A, Haque MA, Baran D (2021). Molecular doping of a naphthalene diimide-bithiophene copolymer and SWCNTs for n-type thermoelectric composites. ACS Appl. Mater. Interfaces.

[46] Freeman DD, Choi K, Yu C (2012). N-type thermoelectric performance of functionalized carbon nanotube-filled polymer composites. PLoS ONE.

[47] Fukui K, Yonezawa T, Shingu H (1952). A molecular orbital theory of reactivity in aromatic hydrocarbons. J. Chem. Phys..

[48] Miller J, Parker AJ (1961). Dipolar aprotic solvents in bimolecular aromatic nucleophilic substitution reactions. J. Am. Chem. Soc..

[49] Saito T, Ohmori S, Shukla B, Yumura M, Iijima S (2009). A novel method for characterizing the diameter of single-walled carbon nanotubes by optical absorption spectra. Appl. Phys. Express.

[50] Tanaka Y (2009). Experimentally determined redox potentials of individual (n,m) single-walled carbon nanotubes. Angew. Chem. Int. Ed..

[51] Han CC, Elsenbaumer RL (1989). Protonic acids: generally applicable dopants for conducting polymers. Synth. Met..

[52] Yurash B (2019). Towards understanding the doping mechanism of organic semiconductors by Lewis acids. Nat. Mater..

[53] Nakashima Y, Nakashima N, Fujigaya T (2017). Development of air-stable n-type single-walled carbon nanotubes by doping with 2-(2-methoxypheny1)-1,3-dimethyl-2,3-dihydro-1*H*-benzo[d]imidazole and their thermoelectric properties. Synth. Met..

[54] Haras M, Skotnicki T (2018). Thermoelectricity of IoT – a review. Nano Energy.

[55] Mai C-K (2015). Varying the ionic functionalities of conjugated polyelectrolytes leads to both p- and n-type carbon nanotube composites for flexible thermoelectrics. Energy Environ. Sci..

[56] Liu WS, Kim HS, Jie Q, Ren ZF (2016). Importance of high power factor in thermoelectric materials for power generation application: a perspective. Scr. Mater..

[57] MacLeod BA (2017). Large n- and p-type thermoelectric power factors from doped semiconducting single-walled carbon nanotube thin films. Energy Environ. Sci..

[58] Zhou WB (2017). High-performance and compact-designed flexible thermoelectric modules enabled by a reticulate carbon nanotube architecture. Nat. Commun..

[59] Bubnova O (2011). Optimization of the thermoelectric figure of merit in the conducting polymer poly(3,4-ethylenedioxythiophene). Nat. Mater..

[60] Menon AK, Meek O, Eng AJ, Yee SK (2017). Radial thermoelectric generator fabricated from n- and p-type conducting polymers. J. Appl. Polym. Sci..

[61] Mukaida M, Kirihara K, Wei QS (2019). Enhanced power output in polymer thermoelectric devices through thermal and electrical impedance matching. ACS Appl. Energy Mater..

[62] Zhou WB (2016). Ultrahigh-power-factor carbon nanotubes and an ingenious strategy for thermoelectric performance evaluation. Small.

[63] Hada M (2019). One-Minute Joule Annealing Enhances the Thermoelectric Properties of Carbon Nanotube Yarns via the Formation of Graphene at the Interface. ACS Appl. Energy Mater..

