# Supplementary material for: Bicyclic-ring base doping induces n-type conduction in carbon nanotubes with outstanding thermal stability in air
Source: Nat Commun. 2022 Jun 20;13:3517. doi: 10.1038/s41467-022-31179-6 (PMC9209455; doi:10.1038/s41467-022-31179-6)
Supplement: Supplementary file 1 — Supplementary Information [file 41467_2022_31179_MOESM1_ESM.pdf]

Supplementary Information

Bicyclic-ring base doping induces n-type conduction in carbon nanotubes with outstanding thermal stability in air

Shohei Horike[,1,2,3,4], Qingshuo Wei[2], Kouki Akaike[2], Kazuhiro Kirihara[2], Masakazu Mukaida[2], Yasuko Koshiba[1,4], and Kenji Ishida[1,4]*

[1]Department of Chemical Science and Engineering, Graduate School of Engineering, Kobe University, 1-1 Rokkodai-cho, Kobe 657-8501, Japan

[2]Nanomaterials Research Institute, National Institute of Advanced Industrial Science and Technology (AIST), 1-1-1 Higashi, Tsukuba 305-8565, Japan

[3]PRESTO, Japan Science and Technology Agency, Kawaguchi 332-0012, Japan

[4]Research Center for Membrane and Film Technology, Kobe University, 1-1 Rokkodai-cho, Kobe 657-8501, Japan

Correspondence to: horike@crystal.kobe-u.ac.jp

Table of Contents

Supplementary Note 1. Molecular Mapping of Dopants
Supplementary Note 2. Chemical Structures of Solvents
Supplementary Note 3. Electrical Conductivity of TBD-doped CNT and Graphite Films
Supplementary Note 4. Measurement Principle of Work Function Shift Using Kelvin Probe
Supplementary Note 5. Raw Data of Thermoelectric Measurements
Supplementary Note 6. List of Chemicals and Materials
Supplementary Note 7. Thermal Diffusivity

Supplementary Note 1. Molecular Mapping of Dopants

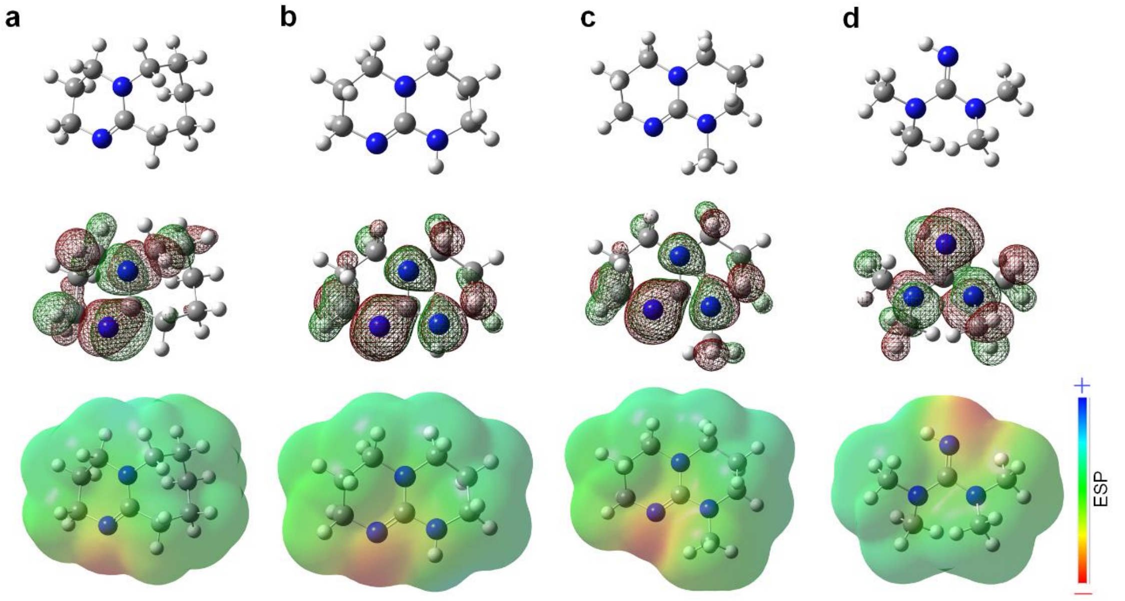

Supplementary Figure 1 DFT (B3LYP/6-31G) calculation results. a DBU, **b** TBD, **c** Me-TBD, and **d** TMG. White, gray, and blue balls indicate hydrogen, carbon, and nitrogen atoms, respectively. Upper panels: optimized molecular structures. Middle panels: HOMO mapping. Red and green colors indicate the positive and negative phases of the orbital, respectively. Lower panels: Electrostatic potential (ESP) mapping. Warm color indicates more negative potential compared to other locations. For all chemicals, the double-bonded nitrogen atom participates in the HOMO and carries a high negative charge, suggesting dominant contribution of this site to the lone-pair electron transfer (electron doping) to CNTs.

Supplementary Note 2. Chemical Structures of Solvents

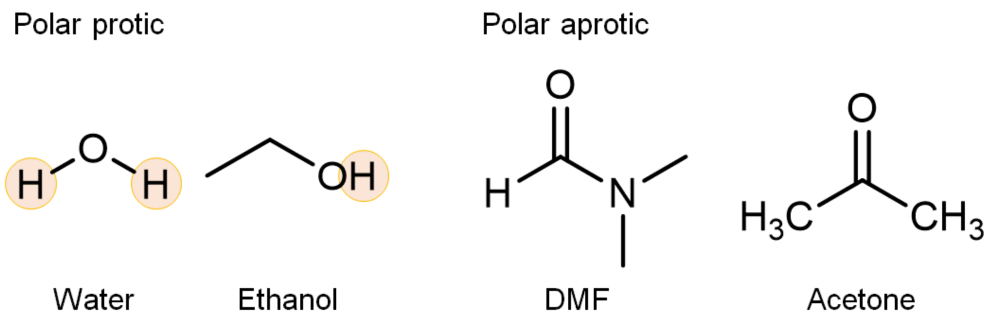

Supplementary Figure 2 Molecular structures of solvents tested for TBD doping to CNTs. (The solvent effects are shown in Fig. 4b of the main article and Supplementary Table 1 of SI). Polar protic solvents contain acidic hydrogens (highlighted in orange), which are expected to accept lone-pair electrons from the base (TBD) to hinder electron transfer from TBD to CNT.

Supplementary Note 3. Electrical Conductivity of TBD-doped CNT and Graphite Films

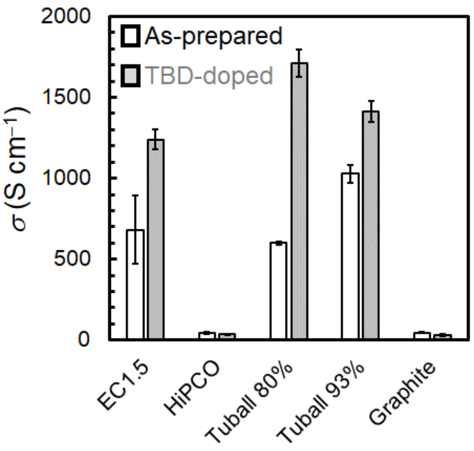

Supplementary Figure 3 Effect of TBD doping on different nanoscale carbon materials in term of electrical conductivity (*σ*) measured at room temperature (~298 K) in air. Doping was performed using DMF solution of TBD (10 mg mL^{-1}). The doping condition was the same as that for single-walled CNTs synthesized using the eDIPS method (EC1.5, Meijo Nano Carbon), which is described in detail in METHOD section of the main article. The percentages (80% and 93%) of the Tuball CNTs indicate purity of the pristine materials. Data statistics: $n \geq 3$. Error bards indicate standard deviation (SD).

Supplementary Note 4. Measurement Principle of Work Function Shift Using Kelvin Probe

Herein, we describe the measurement principle of work function shift, shown in Table 1 of the main article, using Kelvin probe method. First, we consider two different conducting (or semiconducting) materials with different work functions (ϕ_1 and ϕ_2, $\phi_1 \neq \phi_2$). Here, we denotate the energy gaps between the vacuum level and Fermi level of metals and between the vacuum level and the HOMO energy level or the upper end of valence band of semiconductors as the work function. If two conductors are electrically insulated, vacuum level of each material is present at the same energy level, as shown in Supplementary Fig. 4a. Upon creating the interface between these materials without applying external voltage ($V_E = 0$), the vacuum level shifts to generate a contact potential difference ($\Delta V_{21} = (\phi_1 - \phi_2)/e$) so that each Fermi level aligns at the same energy level, as shown in Supplementary Fig. 4b; here, e is the elementary charge. This potential difference is caused by the surface charges that are induced on each material, and thus the interface can be regarded as a capacitor. The accumulated charge (Q) is expressed by the capacitance (C) and the bias ($\Delta V_{21} + V_E$) as:

$$Q = C(\Delta V_{21} + V_{\mathrm{E}}). \qquad (S1)$$

If we periodically change the distance between the materials, the capacitance will change accordingly. Such process will also lead to a periodic change of the surface charge according to Eq. (S1). Time (t) derivative of the surface charge corresponds to the electric current (I) through the external circuit as:

$$I = \frac{dQ}{dt} = \frac{d}{dt} C(\Delta V_{21} + V_{\mathrm{E}}). \qquad (S2)$$

In case of $V_E = 0$, alternating current will be continuously detected during the distance changes between each material. In contrast, the current will be zero when the external voltage of $V_E = -\Delta V_{21}$ is applied (in this case, vacuum and Fermi levels do not shift as shown in Supplementary Fig. 4c). As ΔV_{21} represents the difference of work function of each material, the following relation is immediately obtained when $I = 0$:

$$\phi_2 = \phi_1 + eV_{\mathrm{E}}. \qquad (S3)$$

When a probe with known work function (ϕ_1) is used, we can immediately obtain the absolute value of work function of the sample (ϕ_2) by measuring V_E that can result in zero current. In the present study, we investigated the work function shift ($\Delta\phi^{\mathrm{n-p}}$) of CNTs after TBD doping (the changes in work functions of as-prepared p-type and TBD-doped n-type CNTs). The work function shift was determined using the following equation:

$$\Delta\phi^{\mathrm{n-p}} = \phi_2^{\mathrm{n}} - \phi_2^{\mathrm{p}} = \left(\phi_1 + eV_{\mathrm{E}}^{\mathrm{n}}\right) - \left(\phi_1 + eV_{\mathrm{E}}^{\mathrm{p}}\right) = e\left(V_{\mathrm{E}}^{\mathrm{n}} - V_{\mathrm{E}}^{\mathrm{p}}\right), \qquad (S4)$$

(S5)

where ϕ_2^i is the work function of CNT sample (here, *i* represents n- or p-type polarity), ϕ_1 is the work function of the probe (the identical reference material), and V_E^i is the measured voltage for each CNT sample when $I = 0$ is achieved.

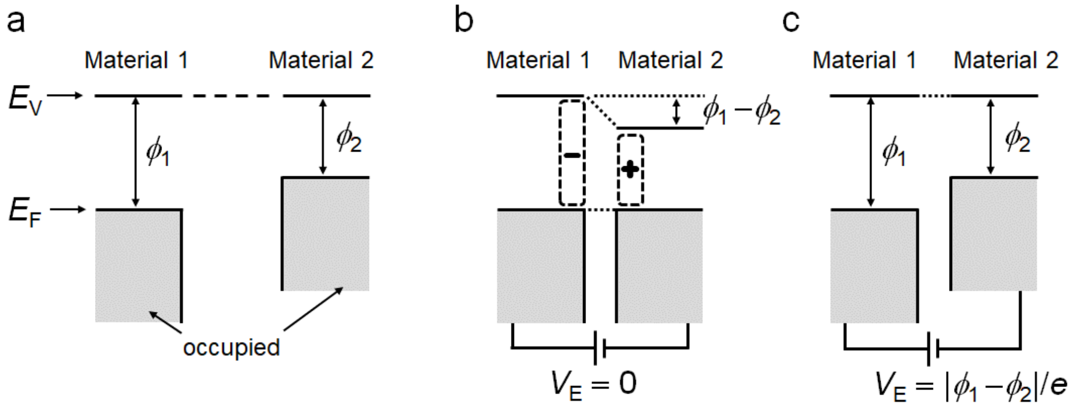

Supplementary Figure 4 Principle of work function measurement using Kelvin probe. Energy diagrams of two metals with different work functions (ϕ_1 and ϕ_2) **a** before contact (under electrical insulation), **b** after contact without bias, and **c** after contact with bias ($V_E = |\phi_1 - \phi_2|/e$, where e is the elementary charge). E_F and E_V represent Fermi level and vacuum level, respectively.

Supplementary Note 5. Raw Data of Thermoelectric Measurements

Supplementary Table 1. Solvent dependence of Seebeck coefficient, electrical conductivity, and power factor of TBD-doped CNT films.*

Solvent	$S^{\dagger}$ [μV K^{-1}]	$\sigma^{\ddagger}$ [S cm^{-1}]	$P^{\S}$ [μW m^{-1} K^{-2}]
Water	−29	740	62
	−24	890	51
	−28	980	76
Ethanol	−32	990	101
	−32	890	91
	−34	860	99
N,*N*-dimethylformamide (DMF)	−32	1160	118
	−30	1310	117
	−29	1190	100
	−28	1310	102
	−34	1220	141
Acetone	−32	1330	136
	−29	1280	107
	−28	1160	90

*Films of EC1.5-CNTs were doped by immersing in TBD solutions with the concentration of 71 mM. The Seebeck coefficients and electrical conductivities in this table are averaged to produce those in Fig. 4b of the main article.

†*S*: Seebeck coefficient; ‡*σ*: electrical conductivity, §*P*: thermoelectric power factor

Supplementary Note 6. List of Chemicals and Materials

Supplementary Table 2. List of chemicals and materials used in this study.

	purity (%)	supplier	remark
1,8-Diazabicyclo[5.4.0]-7-undecene (DBU)	97	FUJIFILM Wako Pure Chemical	
1,5,7-Triazabicyclo[4.4.0]dec-5-ene (TBD)	98.0	Tokyo Chemical Industry	
7-Methyl-1,5,7-triazabicyclo[4.4.0]dec-5-ene (Me-TBD)	95.0	FUJIFILM Wako Pure Chemical	
1,1,3,3-Tetramethylguanidine (TMG)	97	FUJIFILM Wako Pure Chemical	
Deionized water	–	AS ONE	Conductivity ≤ 0.1 mS m^{-1}
Acetone	99	FUJIFILM Wako Pure Chemical	
N, N-dimethylformamide (DMF)	99.5	FUJIFILM Wako Pure Chemical	
Ethanol	99.5	FUJIFILM Wako Pure Chemical	
Brij 30	80	Sigma Aldrich	Nonionic surfactant used for CNT dispersion

Supplementary Table 3. List of carbon nanotube (CNT) samples used in this study.

Sample	purity (%)	supplier	remark
EC1.5	>90	Meijo Nano Carbon	Produced by enhanced direct injection pyrolytic synthesis (eDIPS). EC1.5 (rich in single-walled CNTs). Diameter: 1–3 nm.
HiPCO	– (Residue Fe content < 35 wt%)	Raymor	Raw fluffy powder of single-walled CNTs synthesized using high pressure carbon monoxide (HiPCO). Diameter: ~0.8–1.2 nm.
Tuball (80%)	80	OCSiAl	Tuball. Single-walled CNTs. Diameter: 1.6±0.4 nm
Tuball (93%)	93	OCSiAl	Tuball. Single-walled CNTs. Diameter: 1.6±0.4 nm
s-SWCNT (d = 1.2–1.7 nm)	99.9	NanoIntegris	IsoNanotubes-S. Semiconducting single-walled CNTs. Diameter: 1.2–1.7 nm
m-SWCNT (d = 1.2–1.7 nm)	98	NanoIntegris	IsoNanotubes-M. Metallic single-walled CNTs. Diameter: 1.2–1.7 nm
s-SWCNT (d = 0.7–0.9 nm)	≤ 95 as carbon, ≥ 93 as SWCNT	Sigma Aldrich	Semiconducting single-walled CNTs. (6,5) chirality index. Diameter: 0.7–0.9 nm

Supplementary Note 7. Thermal Diffusivity

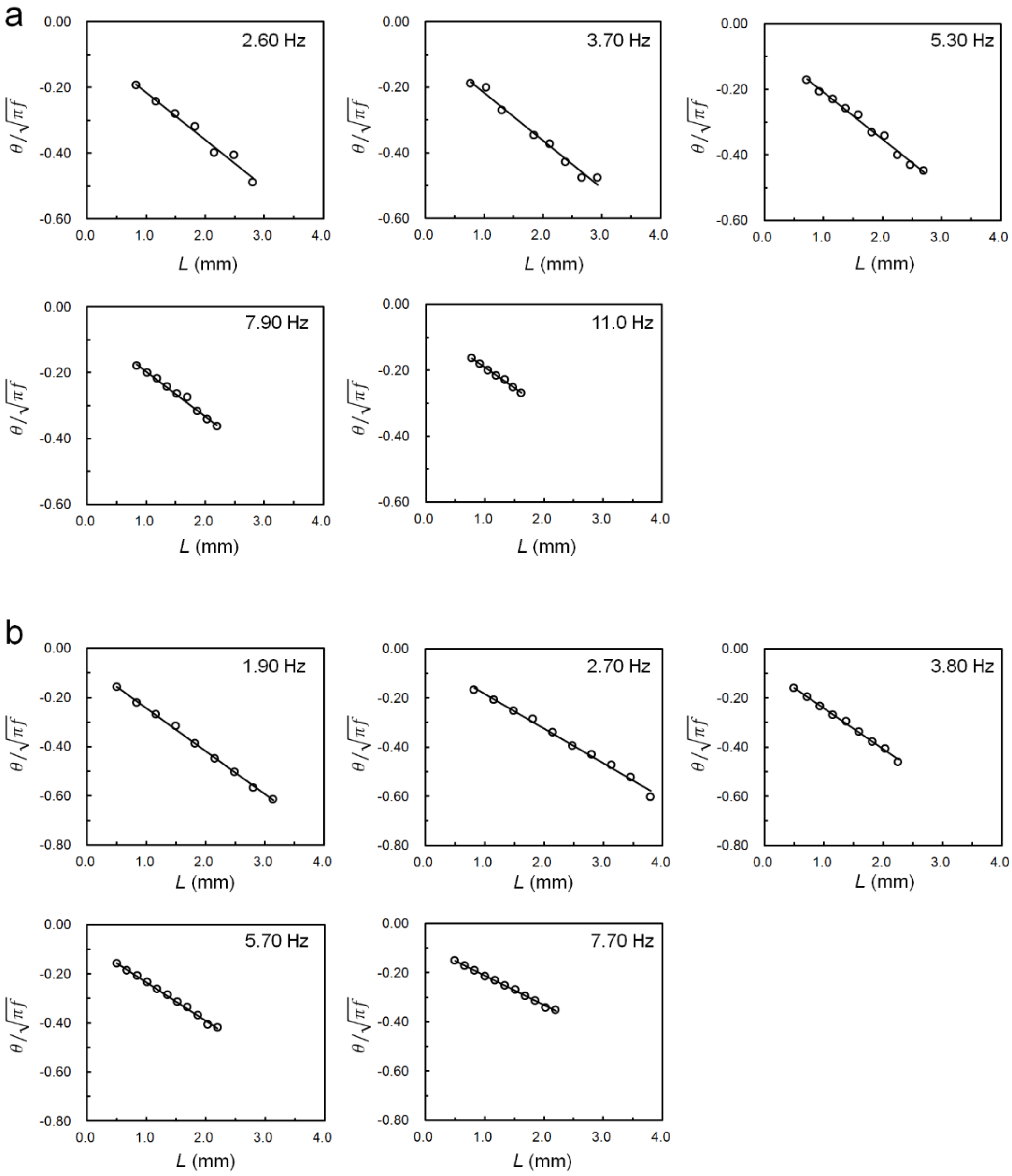

Supplementary Figure 5 In-plane thermal diffusivity. a As-prepared and **b** TBD-doped CNT films. Measurements were performed by the periodic heating radiation thermometry technique. One applies periodic heating to a point on the film surface at varied frequency, while monitoring the phase differences of the radiant temperature oscillation from other points on the specimen. Here, the phase difference (θ) and the heating frequency (f) are related to the distance between the heated and measured spots (L) as well as the thermal diffusivity (a) by $\theta/(\pi f)^{1/2} = -L/a^{1/2}$. The a value was determined from the average slope of the $\theta/(\pi f)^{1/2} - L$ plots with varied f.
